# Beyond Malignancy and Reflux: Laryngeal Tuberculosis Diagnosed in a Patient Presenting With Chronic Dysphonia

**DOI:** 10.7759/cureus.100362

**Published:** 2025-12-29

**Authors:** How Ting Ong, Rachel W Leong, Jereme Y Gan

**Affiliations:** 1 Department of Otorhinolaryngology, Tan Tock Seng Hospital, Singapore, SGP

**Keywords:** case report, dysphonia, extrapulmonary tuberculosis, laryngeal tuberculosis, otorhinolaryngology, tuberculosis

## Abstract

Laryngeal tuberculosis (TB) is a rare extrapulmonary manifestation of TB. Patients often present with non-specific laryngeal complaints and may not have pulmonary TB, which was previously almost always associated with laryngeal TB. This case report describes a patient who initially presented with non-specific symptoms of chronic voice hoarseness and dry cough, who was initially diagnosed with pneumonia. Subsequent otorhinolaryngological evaluation was performed, which showed exudative laryngeal lesions with edema and narrowing of the supraglottis and glottis. The patient underwent urgent fiberoptic intubation to secure the airway, followed by a panendoscopy and biopsy of the laryngeal lesions. Microbiological testing and histopathological examination confirmed the diagnosis of laryngeal TB. The patient was also noted to have pulmonary TB involvement on chest radiographs. The patient was noted to have poorly controlled diabetes mellitus that was newly diagnosed, which is a significant risk factor for laryngeal TB. This case report highlights the ease of misdiagnosis and provides clinicians with a review of the epidemiology, clinical characteristics, diagnostic evaluation, and management of laryngeal TB. Additionally, it draws attention to the possibility of acute airway compromise in laryngeal TB, which is not widely reported in the literature. Laryngeal TB remains a relevant differential diagnosis for patients with chronic laryngeal symptoms, and clinicians should not exclude TB as a diagnosis even in developed or non-endemic regions.

## Introduction

Tuberculosis (TB) is a prevalent disease worldwide that is caused by a group of genetically related mycobacteria collectively termed the *Mycobacterium tuberculosis* complex. The most common causative species is *Mycobacterium tuberculosis*. Initial infection, termed primary TB, usually presents with mild non-specific symptoms, and is typically contained in healthy individuals to become latent TB infection. Most cases of active TB arise due to secondary reactivation of TB, typically triggered by an immunocompromised state. Most patients with active TB present with pulmonary disease, although an estimated 15-20% of patients present with extrapulmonary TB [1].

Laryngeal TB was a common manifestation of extrapulmonary TB in the early twentieth century. However, with the advent of anti-TB chemotherapy, it has since become a rare entity seen in fewer than 1% of patients with TB [2]. Given the relative paucity of disease, clinicians may not consider laryngeal TB in their differential diagnoses for patients presenting with laryngeal symptoms, especially in non-endemic or developed regions with low rates of TB.

While TB is endemic to Singapore, it is a developed nation that sees low rates of TB, with a nationwide total of 1,156 patients newly diagnosed with active TB in 2024. Of these patients, 180 (15.5%) were diagnosed with extrapulmonary infection. We present a case of one such patient who presented with non-specific laryngeal symptoms and was eventually diagnosed with laryngeal TB with pulmonary involvement. This case report highlights the importance of considering laryngeal TB as a differential diagnosis for patients with chronic laryngeal complaints, even in a developed country with a low rate of TB, and discusses the diagnostic challenges and considerations behind the management of laryngeal TB.

## Case presentation

A 38-year-old Filipino female presented to the emergency department with complaints of worsening sore throat and voice hoarseness associated with chronic cough for the past seven weeks. This was associated with low-grade fever and anorexia for the past four days. She did not report any recent travel history, known sick contacts, or symptoms of gastroesophageal reflux disease. She was a non-smoker. She had previously visited a general practitioner and was treated with a course of oral amoxicillin/clavulanic acid, with no significant improvement in symptoms. She did not report any loss of weight or night sweats.

**Figure 1:**
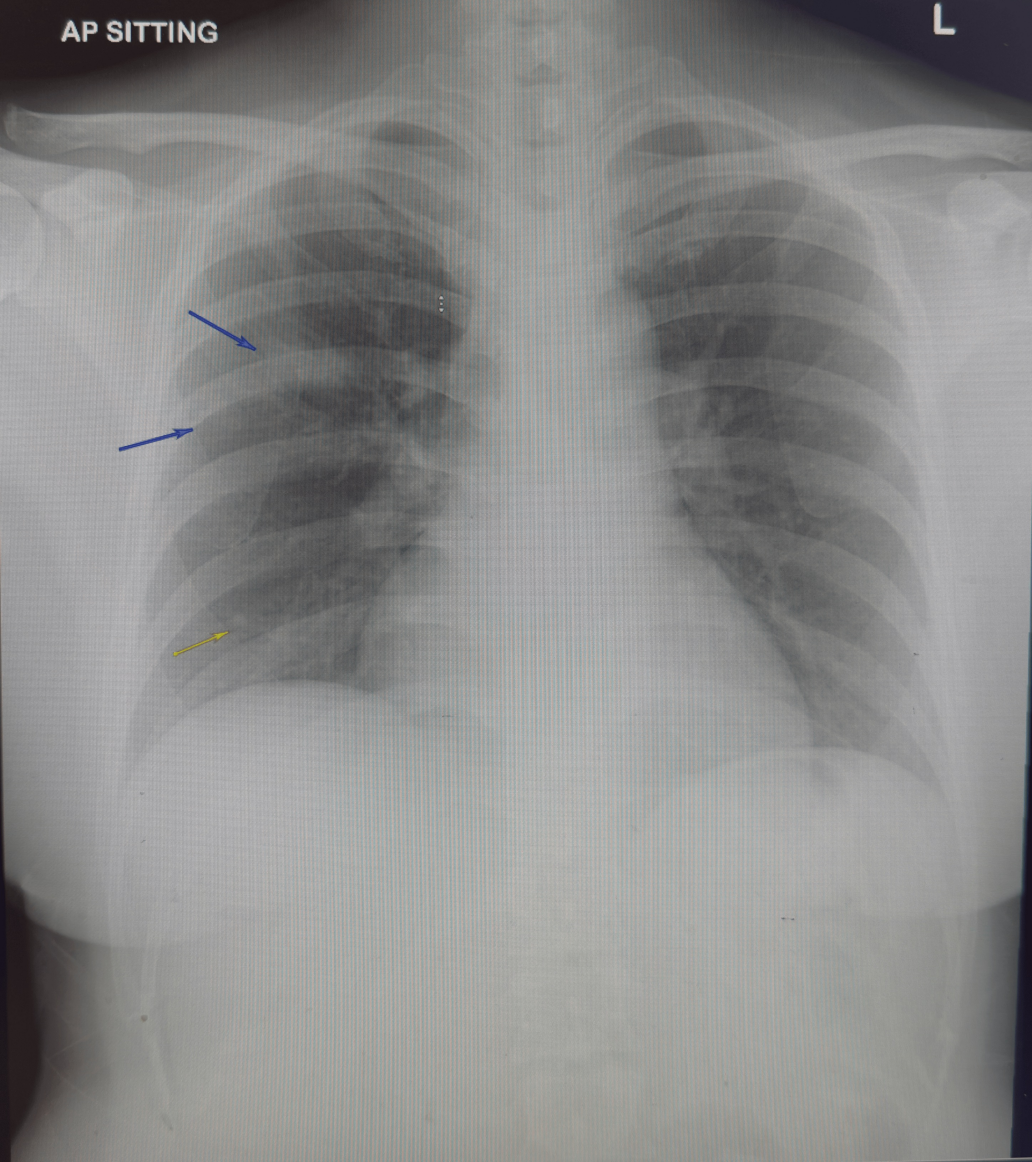
Chest X-ray performed on admission showing right mid-zone opacities.

The patient’s voice was noted to be hoarse and raspy. Examination of the oral cavity showed pharyngeal erythema. Auscultation of the lungs was clear, and there was no cervical lymphadenopathy. The remainder of the physical examination was unremarkable. Initial laboratory investigations on admission revealed anemia, neutrophilia, lymphopenia, and elevated inflammatory markers (Table 1). Chest radiographs showed focal opacities over the right mid-zone (Figure 1). The patient was diagnosed with community-acquired pneumonia and started on antibiotic treatment with intravenous amoxicillin/clavulanic acid and oral azithromycin. She was admitted to the General Medicine service overnight.

**Table 1:** Laboratory investigations on admission.

Investigations	Values	Reference values
White blood cells	9.3 × 10^9^/L	4.0–9.6 × 10^9^/L
Neutrophils	8.29 × 10^9^/L	1.90–6.60 × 10^9^/L
Lymphocytes	0.63 × 10^9^/L	1.10–3.10 × 10^9^/L
Hemoglobin	8.6 g/dL	11.8–14.6 g/dL
C-reactive protein	66.3 mg/L	0.0–5.0 mg/L
Procalcitonin	0.11 µg/L	0.00–0.06 µg/L

**Figure 2:**
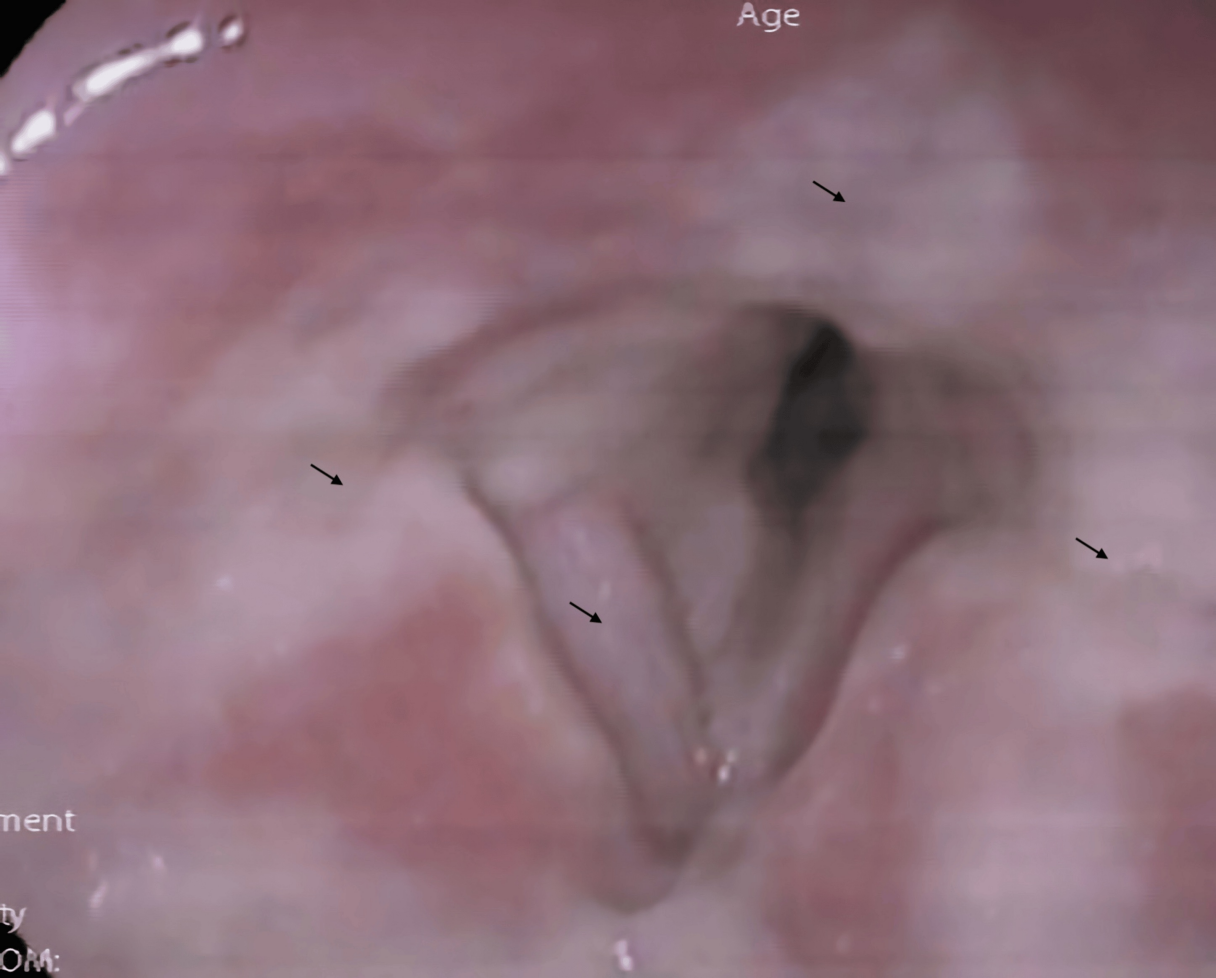
Nasoendoscopy showing exudative, non-ulcerative lesions over the supraglottis and glottis.

Additional investigations with urinary legionella and pneumococcal antigen assays, influenza polymerase chain reaction (PCR), and COVID-19 PCR tests were obtained the next day. Otorhinolaryngology was also consulted for evaluation of her dysphonia. Inspiratory stridor was noted on review by Otorhinolaryngology. Flexible nasoendoscopy showed widespread exudative, non-ulcerative lesions over the supraglottis and glottis with edema and narrowing of the laryngeal inlet (Figure 2). Concerns were raised for possible laryngeal TB given the clinical and radiological findings. The examining otorhinolaryngologist was also concerned for potential impending airway compromise given the clinical findings of stridor and a significantly narrowed upper airway on laryngoscopic examination. An Anaesthesia consult was obtained, with the decision made to proceed with urgent awake fiberoptic intubation (AFOI) to secure the patient’s airway before performing a panendoscopy for biopsy of the laryngeal lesions. AFOI with a size 5 endotracheal tube was performed successfully before the induction of general anesthesia. Intraoperatively, exudates were seen over the laryngeal surface of the epiglottis, ventricular folds, and vocal cords, with extension to the third and fourth tracheal rings. No lesions were found on rigid esophagoscopy. Multiple biopsies were taken and sent for histopathological examination, aerobic and anaerobic bacterial cultures, fungal smears and cultures, acid-fast bacilli (AFB) smears and cultures, and *Mycobacterium tuberculosis* complex PCR testing. The patient was kept intubated and admitted to the Intensive Care Unit postoperatively. Sputum and endotracheal aspirates were sent for AFB smears and cultures as well.

**Figure 3:**
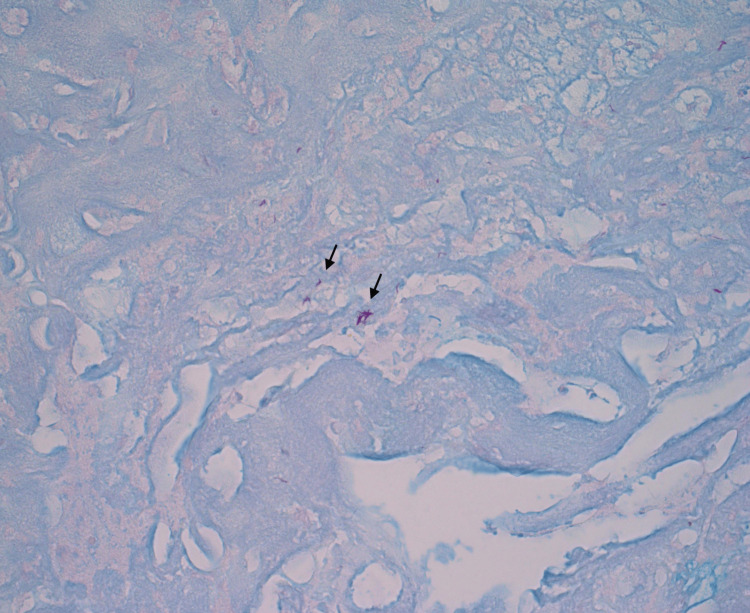
Ziehl-Neelsen stain of laryngeal tissue showing the presence of acid-fast bacilli under 40× magnification.

Histopathological examination revealed an acute-on-chronic inflammatory infiltrate with positive Ziehl-Neelsen staining for AFB (Figure 3), with no evidence of viral inclusions, dysplasia, or malignancy. *Mycobacterium tuberculosis* PCR from biopsy specimens and AFB smears from sputum and endotracheal aspirates were positive. Point-of-care testing for drug susceptibility was done using the GeneXpert MTB/RIF Ultra assay, which did not detect rifampicin resistance. Urinary legionella and pneumococcal antigen tests, influenza PCR, and COVID-19 PCR were all negative. Respiratory Medicine was consulted, and anti-TB chemotherapy with rifampicin, isoniazid, pyrazinamide, and ethambutol was initiated. Intravenous dexamethasone was started to improve airway swelling and the chances of a successful trial of extubation. The patient was extubated successfully on postoperative day one, following a positive leak test. Intravenous dexamethasone was continued at tapering doses over the next few days. A repeat flexible nasoendoscopy performed after extubation showed marked improvement in upper airway edema with some remaining exudates, and the patient reported an improvement in her sore throat and dysphonia. Further investigations revealed poorly controlled diabetes with glycated hemoglobin of 11.6%. The human immunodeficiency virus (HIV) screen was negative. The patient was started on oral hypoglycaemic agents to optimize her glycemic control. She was discharged well after a total inpatient stay of five days.

The patient was kept on directly observed therapy and given a follow-up appointment two weeks after her discharge at the National Tuberculosis Care Centre in Singapore. As the patient was employed as a live-in domestic worker, close household contacts were identified and screened separately under the National Tuberculosis Programme at the National Tuberculosis Screening Centre. At the two-week review, the patient remained compliant with anti-TB chemotherapy and was noted to have complete resolution of her sore throat, cough, and dysphonia. An outpatient Otorhinolaryngology review was scheduled one month after her discharge, but the patient was repatriated to the Philippines before attending the appointment.

## Discussion

The disease burden of TB remains high worldwide, and it is also estimated to be the world’s leading cause of death from a single infectious agent, replacing COVID-19 in recent years [3]. The incidence of upper respiratory tract TB, including laryngeal TB, has also increased, associated with risk factors of HIV infection, diabetes, smoking, malignancy, and immunosuppressive drugs [4,5].

Historically, the majority of laryngeal TB cases have concomitant pulmonary disease, with laryngeal involvement arising due to bronchogenic spread of TB bacilli from the lungs. This is defined as secondary laryngeal TB. However, changes in the clinical patterns of laryngeal TB have been noted. Laryngeal TB was previously seen in younger patients with pulmonary TB, aged 20 to 30, who presented with symptoms of dyspnea and constitutional symptoms of fatigue, weight loss, night sweats, and fever. Today, it tends to involve older patients aged 40 to 60, who present with laryngeal symptoms of hoarseness, odynophagia, and dysphagia [6]. Studies have reported up to 40.6% of patients with laryngeal TB who do not have evidence of pulmonary disease [7], which is defined as primary laryngeal TB and is thought to arise from direct seeding from inhaled TB bacilli, or from hematogenous or lymphatic spread. Interestingly, our patient was diagnosed with secondary laryngeal TB, but presented with laryngeal symptoms rather than the expected pulmonary and constitutional symptoms associated with pulmonary TB. This has been corroborated by a recent literature review on laryngeal TB, which found constitutional symptoms in only 41% of patients [8]. Instead, patients with laryngeal TB present mostly with dysphonia, followed by dysphagia, odynophagia, and cough [4,8,9]. Clinicians should recognize that the lack of classical constitutional TB symptoms does not rule out TB in patients presenting with laryngeal symptoms.

Laryngoscopic examination should be performed in patients with chronic voice hoarseness lasting for 15 days or longer, and especially if there is clinical suspicion of laryngeal TB. Involvement of the posterior laryngeal structures was previously pathognomonic for laryngeal TB, but there is now a predilection for anterior sites such as the true vocal folds and epiglottis [4,5,8,10,11]. Morphologically, lesions can take on a wide variety of appearances, which are commonly classified as granulomatous, ulcerative, polypoid, and non-specific inflammatory lesions [4,5,12]. Our patient presented with non-specific whitish exudative and edematous lesions, which are comparable to classical descriptions of laryngeal TB lesions [11]. Nevertheless, studies suggest that hypertrophic or exophytic lesions have become predominant, which can mimic laryngeal malignancy instead [12,13]. The potential for misdiagnosis given the wide variety of appearances on laryngoscopic examination is evident, with clinicians commonly diagnosing patients with laryngeal malignancy, leukoplakia, or laryngitis from laryngopharyngeal reflux on initial consultation [14].

Evaluation of suspected laryngeal TB should include adjunct investigations such as AFB smears and cultures, bacterial cultures, and TB PCR analysis of tissue or fluid samples [15]. Chest radiographs are helpful as an initial screen for concomitant pulmonary disease, and CT scans can also further support the diagnosis of laryngeal TB. Characteristically, patients with laryngeal TB have CT findings of bilateral diffuse laryngeal lesions with preservation of laryngeal architecture [16]. While bacterial cultures remain the gold standard for the diagnosis of TB [15], biopsies and histopathological examination remain crucial in the diagnosis of laryngeal TB and help rule out other differentials such as laryngeal malignancy [14]. Given the time required for bacterial cultures, histological evidence of caseating granulomas is often considered sufficient to initiate anti-TB therapy in clinical practice [4]. Treatment of laryngeal TB is with standard anti-TB chemotherapy, and patients typically respond well within weeks, accompanied by normalization of laryngeal morphology and improvement of vocal symptoms [4,8,12]. With rising rates of multi-drug-resistant TB (MDR-TB), rapid molecular drug susceptibility testing for resistance to rifampicin with or without isoniazid is recommended where available to detect MDR-TB [15]. While the management of MDR-TB is beyond the scope of this case report, clinicians should refer to established guidelines for drug-resistant TB regimens and consider consulting an infectious disease or TB expert [17].

The presence of stridor in our patient prompted concern for subacute airway obstruction and led to the decision to pre-emptively secure the airway via fiberoptic intubation. When not treated early, laryngeal TB is known to cause complications of posterior glottic stenosis, subglottic stenosis, muscular involvement, and vocal cord paralysis, leading to airway obstruction that may necessitate tracheostomy [18]. While rare, patients can present with acute airway obstruction requiring emergency airway management techniques and even tracheostomy, as described by Cole et al. in a case report [19]. In such cases, clinicians would benefit from knowledge of the Difficult Airway Society guidelines to guide approaches to airway management, and should consider early multidisciplinary involvement of Anesthesia, Otorhinolaryngology, and Intensive Care physicians.

Finally, given the transmissibility of TB, the identification and screening of close patient contacts is critical to stop further spread of the disease. National TB screening programmes, such as the one present in Singapore, can be helpful to consolidate efforts for screening and management of close contacts should they develop infection. Further global efforts are crucial to ensure equitable access to screening for contacts and high-risk groups, which is defined as a key pillar to the End TB Strategy by the World Health Organization [3].

## Conclusions

TB has often been described as the great mimicker with its variety of clinical presentations. Although relatively rare in this day and age, laryngeal TB lives up to this reputation, and there appears to be a resurgence of disease associated with changing patient demographics and clinical patterns. This case report presents several key takeaways to help clinicians avoid possible pitfalls when managing patients with laryngeal TB. First, clinicians should recognize that patients with laryngeal TB can present with only laryngeal complaints, such as cough and chronic dysphonia, with a higher proportion of these patients having primary laryngeal TB without pulmonary involvement. The lack of constitutional symptoms of TB, such as weight loss, anorexia, and night sweats, does not rule out the possibility of TB infection of the larynx. A high degree of suspicion is needed to avoid ruling out the disease prematurely during initial evaluation. Second, a combination of clinical, microbiological, histological, and radiological investigations is required to clinch the diagnosis of laryngeal TB. Laryngoscopic evaluation alone is not sufficient, with high rates of misdiagnosis based on the morphological appearance of the lesions. Obtaining samples for microbiological testing, along with histopathology, is recommended to definitively diagnose laryngeal TB, while enabling clinicians to also evaluate for significant differentials such as malignancy. Lastly, we draw attention to the airway management techniques used in the management of our patient. While acute airway compromise remains a rare complication of laryngeal TB, it is important to recognize signs of potential airway obstruction. Inspiratory stridor was the only bedside clinical sign present in our patient, and the extent of glottic swelling and edema was only identified on laryngoscopic examination one day after admission. Early and timely specialist input should be sought in suspected cases of airway compromise, especially during the initial evaluation.
